# Globe Rupture and Protrusion of Intraocular Contents from Fall in Elderly Patient

**DOI:** 10.7759/cureus.5988

**Published:** 2019-10-24

**Authors:** Andrew Hanna, Rohan Mangal, Tej G Stead, Latha Ganti

**Affiliations:** 1 Emergency Medicine, Graduate Medical Education, University of Central Florida, Orlando, USA; 2 Emergency Medicine, Johns Hopkins University, Baltimore, USA; 3 Emergency Medicine, Brown University, Providence, USA; 4 Emergency Medicine, Envision Physician Services, Orlando, USA

**Keywords:** emergency medicine, ophthalmology, trauma, globe rupture

## Abstract

The authors present a case of globe rupture from a fall in an elderly patient. This patient had her intraocular contents protruding and experienced complete vision loss in her right eye. The emergency management and downstream surgical care is discussed, as well as the use of the Ocular Trauma Score to predict prognosis. Our patient had an Ocular Trauma Score of 1, considering right retinal detachment and perforating injury.

## Introduction

Amongst serious eye injuries, 40% are attributable to penetrating and perforating injury [1]. Globe rupture occurs when the structure of the cornea or sclera is disrupted, usually due to trauma. Symptoms of globe rupture include eye deformity, eye pain, and vision loss. Sometimes, if blunt force directly impacts the eye, the sclera may rupture due to intraocular pressure. Globe injuries are relatively uncommon, with an incidence of 3.5 per 100,000 eye injuries [2]. In an elderly population, such injuries most commonly occur due to falls, the leading cause of injury in the geriatric population [3]. We present an unusual case of globe rupture with protruding intraocular contents following a fall incident in an elderly patient.

## Case presentation

**Figure 1:**
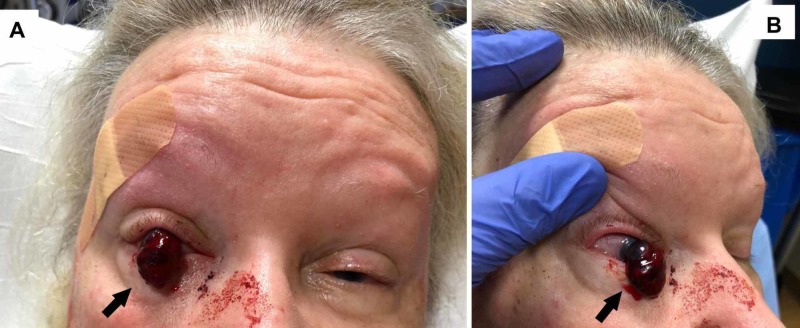
A, Front view, B lateral view depicting globe rupture with extrusion of eye contents (arrows).

A 75-year-old female presented to the emergency department with a ruptured right globe with expulsive choroidal hemorrhage following a ground level fall, in which the patient’s glasses shattered into her right eye. The patient’s intra-ocular contents were extruded with a 1 cm hematoma protruding anteriorly and inferiorly from the right globe (Figure 1A, 1B). She reported complete vision loss and no light perception (NPL) in the right eye with moderate pain. Notably, the patient has previously had four cataract surgeries in her right eye. Medical attention was not sought out immediately, but delayed until the following morning when the patient reported a mass protruding from her eye, which she noticed while eating her breakfast. The patient lived alone, which is presumably why no one could tell her that her eye needed attention.

**Figure 2:**
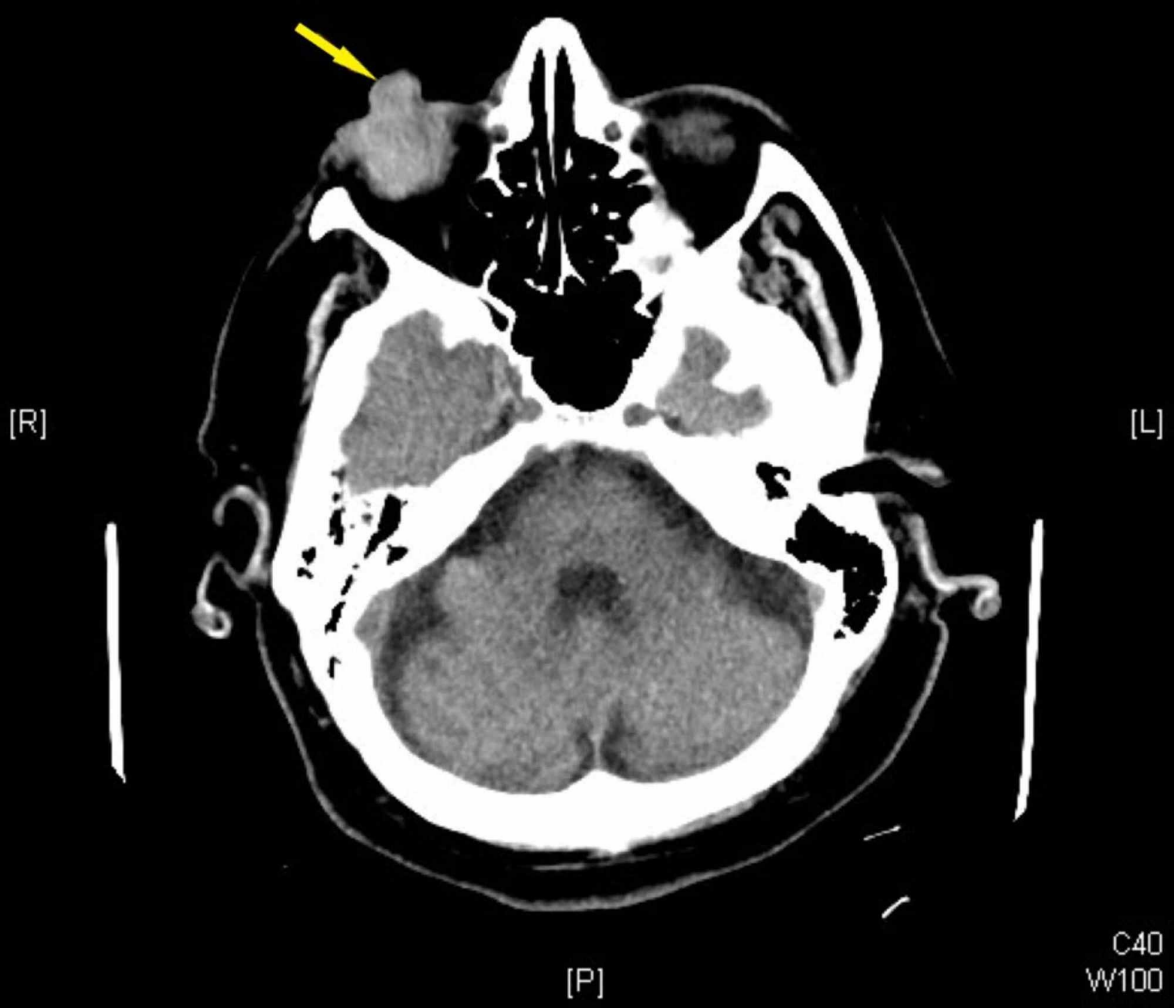
CT demonstrating extrusion of eye contents from right globe (arrow).

A computed tomography scan confirmed protrusion of intraocular contents from the globe (Figure 2). The eye was patched, and trauma surgery and ophthalmology were consulted. The patient received intravenous morphine and ondansetron for analgesia and antiemetic. Her vital signs and mental status remained stable and at baseline throughout her emergency department course.

The patient’s right eye could be repaired cosmetically, but not functionally. After discussion and consent by the patient and the family, the patient underwent primary enucleation. The patient did not suffer any injury to the left eye from the incident. Following surgery, she was transferred to a rehabilitation center.

## Discussion

Emergency management of globe rupture includes preventing an increase in intraocular pressure by providing analgesia and antiemetics, patching the eye to prevent pain and avoid contamination, and prompt ophthalmologic consultation.

Beyond emergency department management, closure of the wound is usually recommended as soon as possible. Surgical options for definitive repair include enucleation and evisceration. Enucleation describes complete removal of the eye, whereas evisceration removes intraocular contents while retaining the scleral shell. The decision of which procedure to perform lies with the surgeon and patient. The main reason advanced for performing enucleation is to prevent sympathetic ophthalmia (SO) [4], which presents as a bilateral diffuse uveitis. Patients report insidious onset of blurry vision, pain, epiphora, and photophobia in the sympathizing, non-injured eye [5]. It is also thought that if the patient has NPL, then it is futile to attempt secondary repair.

However, a review of the US Eye Injury Registry reports that 13% of cases with NPL demonstrate improvements in vision from secondary repair upon patient follow-up, and thus a delayed approach may be better [4]. Furthermore, the fear of SO may be overstated. A large-scale review reported minimal risk of SO following evisceration [6]. In general, evisceration is an easier surgical procedure and is generally preferred by patients for providing greater motility with a better cosmetic appearance. However, enucleation is generally less susceptible to serious complications and has become more cosmetically similar to eviscerations with improved surgical techniques [7]. A review of 107 patients who underwent enucleation or evisceration supports enucleation as a last resort in situations where a patient’s eye is beyond repair and has blindness with complete disfiguration [8].

One prognostic tool that may be helpful is the Ocular Trauma Score (OTS), which estimates visual prognosis six months after an injury. This score was derived from a registry of over 2500 patients in the United States and Hungary, and is based on the presence of the following variables: visual acuity, globe rupture, endophthalmitis, perforating injury, retinal detachment, and relative afferent pupillary defect [9]. The OTS of this patient was 1 with NPL, perforating injury, and retinal detachment. An OTS of 1 denotes that the probability of the patient’s final outcome remaining NPL is 73%, and the likelihood of light perception is only 17% at follow-up. This prognosis may have factored into the patient's ophthalmologist's surgical approach.

## Conclusions

In cases of globe rupture, surgical repair options include enucleation and evisceration. Enucleation may be better suited for patients with severe, irreparable trauma to the eye, with no potential for improvement. However, evisceration may be preferred for cosmetic reasons. Sympathetic ophthalmitis is a potential outcome for patients with penetrating trauma to the eye that is more likely to occur with time. To avoid complications, a timely decision for surgery is strongly recommended. The OTS may be helpful in guiding the approach to surgical repair.
